# Supplementary material for: Second Generation DNA Methylation Age Predicts Cognitive Change in Midlife: The Moderating Role of Childhood Socioeconomic Status
Source: Res Sq. 2024 Dec 24:rs.3.rs-5551592. Preprint. [Version 1] doi: 10.21203/rs.3.rs-5551592/v1 (PMC11703339; doi:10.21203/rs.3.rs-5551592/v1)
Supplement: Supplement 1 [file NIHPPRS5551592v1-supplement-1.pdf]

Supplementary Files

This is a list of supplementary files associated with this preprint. Click to download.

- SciReportsSIDNAmAge.docx
